# Insights on the process to develop Australia’s first national climate risk assessment

**DOI:** 10.1016/j.isci.2025.112068

**Published:** 2025-02-20

**Authors:** Fanny A. Boulaire, Stephen Cook, Aysha Fleming, Lygia Romanach, Tim Capon, Murni Po, Rebecca Darbyshire, Guy Barnett, Sonia Bluhm, Brenda B. Lin

**Affiliations:** 1 CSIRO Environment, Dutton Park, QLD 4102, Australia; 2 CSIRO Environment, Darwin, NT 0828, Australia; 3 CSIRO Environment, Hobart, TAS 7005, Australia; 4 CSIRO Environment, Black Mountain, ACT 2601, Australia; 5 CSIRO Agriculture and Food, Black Mountain, ACT 2601, Australia; 6 Scientell, 8 La Trobe St, Melbourne, VIC 3000, Australia

**Keywords:** Earth sciences, Climatology, Environmental policy

## Abstract

Countries are undertaking national climate risk assessments to help decision-makers respond effectively to climate change impacts. Australia has also started this process, with the release in early 2024 of the first pass assessment report of its National Climate Risk Assessment.

This paper describes our experiences of the process undertaken in Australia to conduct a first pass qualitative assessment of climate risks and compare it with the learnings gathered from a desktop review of 15 other national climate risk assessments. Highlighting similarities and differences in approaches, this paper offers insights for others embarking on a similar journey or improving existing ongoing processes. It identifies four process themes that could contribute to a common framework for these assessments, while acknowledging the need for tailored approaches. Having a common framework could increase awareness and incentives for international collaboration on common or shared risks, and lead to more coordinated climate mitigation and adaptation actions.

## Graphical abstract

**Figure:**
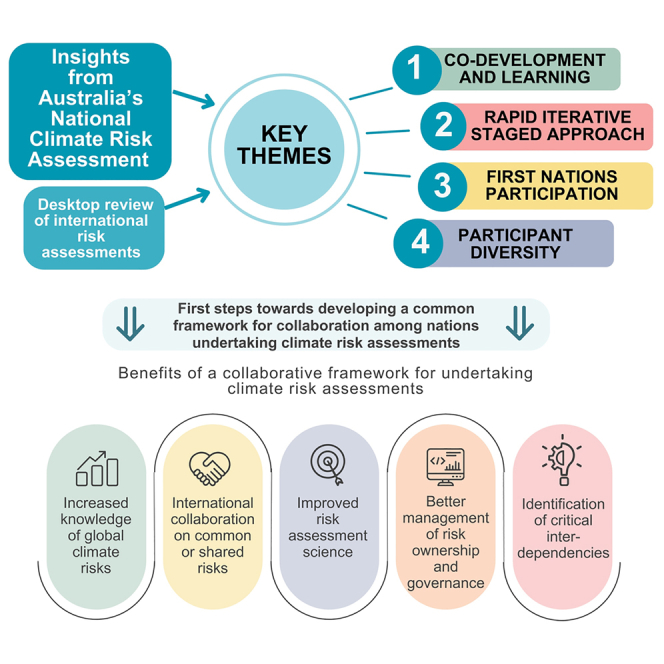

## Introduction

Climate change presents risks that impact society, the environment, industry, and government.^1^^,^^2^ Despite efforts to mitigate climate change impacts by reducing greenhouse gas emissions, an increase in the frequency and intensity of extreme climate events has already been observed, indicating the need for rapid and effective adaptation actions.^3^^,^^4^^,^^5^ With growing awareness and concerns in Australia and around the world, many industries, community groups and multiple levels of government have started their journey to build climate resilience and better prepare for climate-related disasters and recovery. Some governments and organizations have developed their own regional or sector-based climate risk assessments to identify their climate risks and to inform the development of adaptation plans to manage the impacts. In Australia, for example, organizations such as the Climate Council of Australia^6^ and the Reserve Bank of Australia^7^ have undertaken climate risk assessments. Each state and territory government in Australia has undertaken some form of climate risk assessment,^8^ while almost one-fifth of all Australian local governments have declared a climate emergency and are considering their climate risks.^9^ At sector level, national approaches include the National Statement on Climate Change and Agriculture^10^ and National Health and Climate Strategy,^11^ developed in collaboration with the states and territories and drawing on existing work undertaken in these jurisdictions.^12^^,^^13^^,^^14^^,^^15^ While regional or sector-based analyses are important, regions and sectors often depend on one another, leading to interrelated risks that span geographies and economies.^16^ Critical national infrastructure systems, such as energy, transport, and built infrastructure, as well as financial and health systems, provide essential services for communities and businesses. While decisions or management of these systems might be made at the state or regional level, they usually have their policies determined at the national level and require the coordination of multiple stakeholders to protect their operation and function. This is especially important when climate-related disasters occur on large scales and impact multiple jurisdictions.^17^ Comprehensive national strategies are typically required to protect the natural, social, and economic assets that a country relies on for its sustainability and resilience. National climate risk assessments are often a crucial step in developing and evolving coordinated national strategies to address climate change, facilitating the discussions across regions and sectors required to reveal the systemic nature of climate risk.

Many governments around the world (e.g., the UK, Germany, the US, Canada, and New Zealand) have undertaken national assessments of their climate risks. This is often a response to national obligations to manage climate change impacts and extreme events,^18^^,^^19^^,^^20^^,^^21^^,^^22^^,^^23^ and to develop and implement appropriate adaptation plans.^24^^,^^25^ Australia commenced its first National Climate Risk Assessment (NCRA) in 2023 to identify the priority risks from climate change and inform the development of a National Adaptation Plan.^26^ In this paper, we (the authors were key contributors to the first pass of the Australia’s NCRA; roles included project development and management, domain expertise, and facilitation) describe our experience with the process used to deliver the first pass of Australia’s NCRA, which we contrast with the findings from a desktop review of national climate risk assessments undertaken in other countries. Drawing on our experience when undertaking the first pass of Australia’s NCRA we developed a list of criteria for information to collect and compare across national climate risk assessments. We provide perspectives on the learnings from national climate risk assessments and highlight similarities and differences that are important to consider for future climate risk assessments.

While standardizing climate risk assessments might not be applicable to all countries, and is not the objective of this paper, opportunities exist to look across the multiple approaches taken by countries to improve the outcomes and efficiency of their process. If climate risk assessment processes can be more aligned and systematic, there is greater potential for countries to work together, learn from each other and develop monitoring and evaluation frameworks that work globally to support climate risk discussions at international forums. This would assist in achieving better and more unified decisions and in the implementation of effective coordinated actions.

## The first pass of Australia’s NCRA

The methodology for Australia’s first NCRA was published in 2023^8^ and is a systems-based approach that involves two stages. A rapid first pass qualitative assessment to identify priority climate risks to the nation, followed by a more detailed second pass quantitative assessment of the priority risks to inform development of a National Adaptation Plan.^26^ The NCRA methodology was based on previous work and guidance from the Intergovernmental Panel on Climate Change (IPCC) and more recent climate risk assessment frameworks that consider complex risk.^27^^,^^28^

This paper is focused on the authors’ experiences with the process of implementing the first pass of the NCRA in the second half of 2023. The process was organized by 8 systems of national significance, as defined in the NCRA methodology.^8^ These 8 systems are listed as follows.

• Health and Social Support;
• Primary Industry and Food;
• Infrastructure and Built Environment;
• Natural Environments;
• Defense and National Security;
• Regional and Remote Communities;
• Economy, Trade and Finance; and
• First Nations Values and Knowledge.

**Figure 1:**
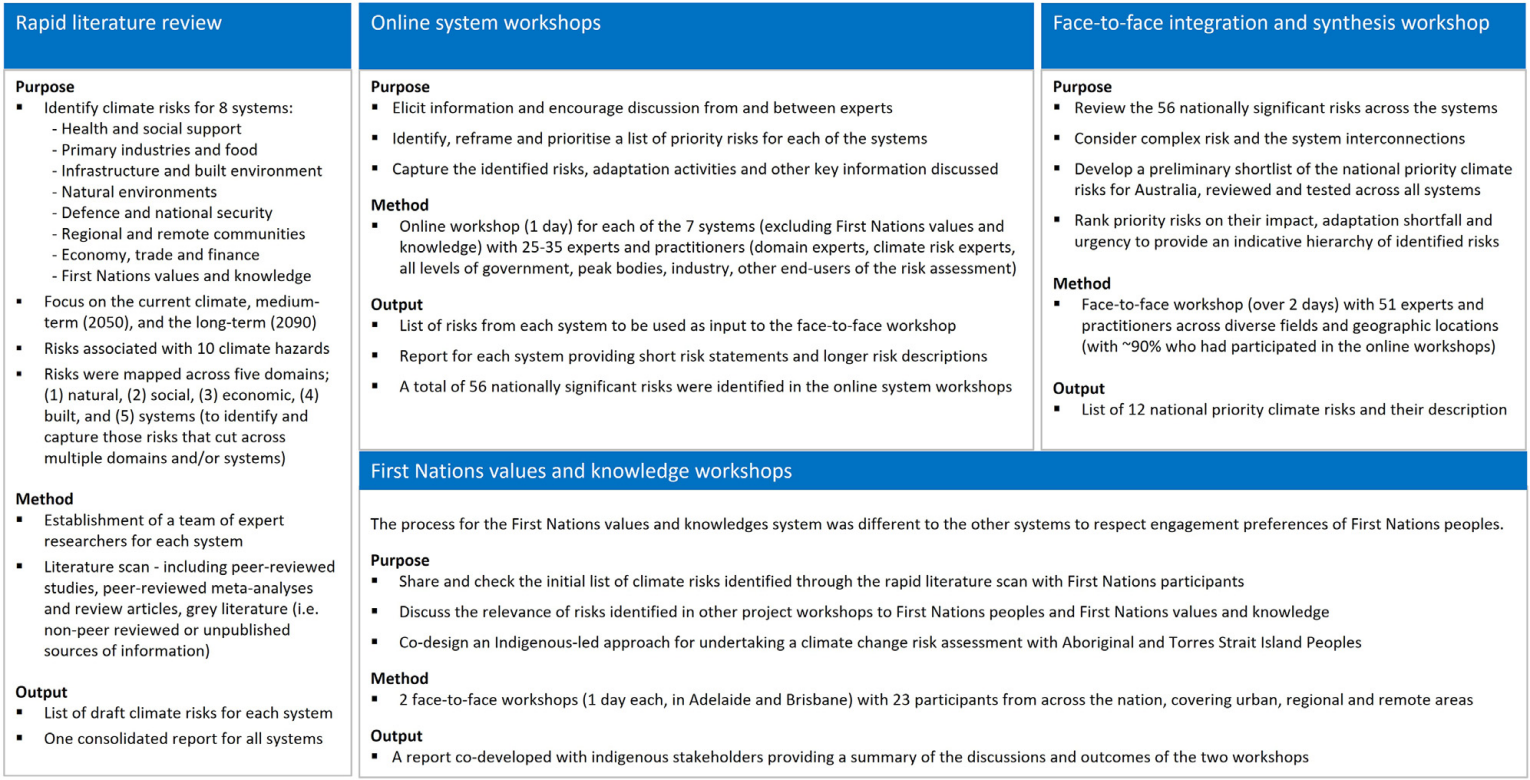
The different steps involved in the first pass of the Australian NCRA, where the goal was to identify priority risks for quantitative assessment in the second pass. It comprised a rapid scan of literature, 7 online system workshops, a face-to-face integration and synthesis workshop, and two workshops on First Nations Values and Knowledge.

An overview of the process used to deliver the first pass of the NCRA is provided in Figure 1.

**Figure 2:**
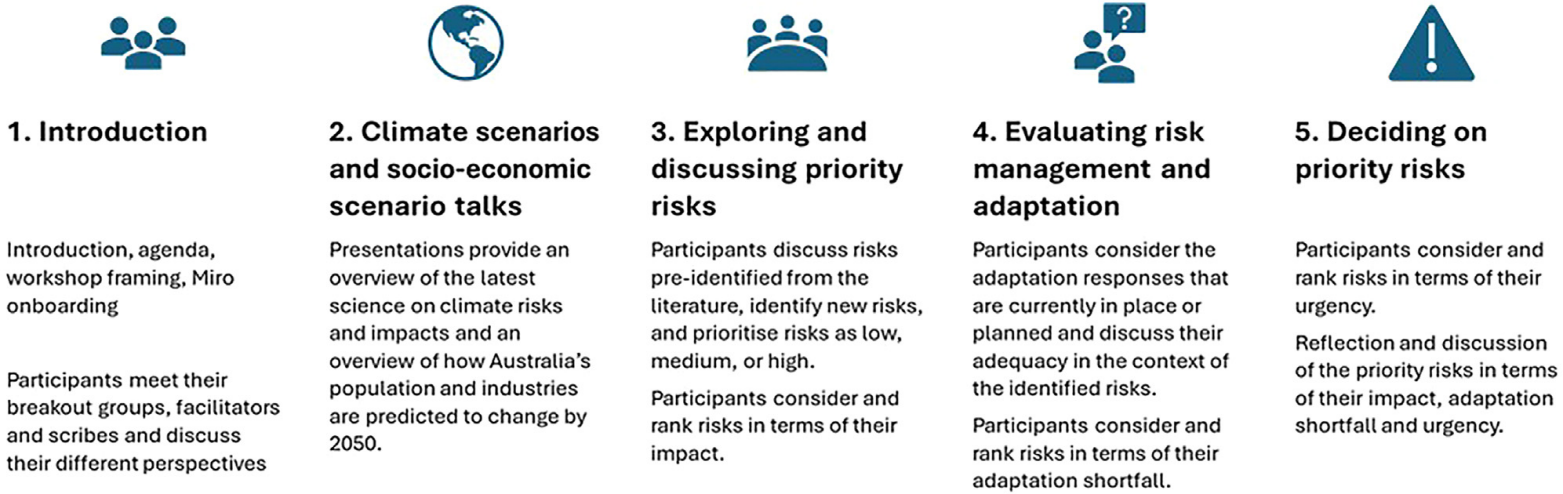
Overview of the online system workshop process implemented as part of the first pass of the NCRA

For each system, a literature review was undertaken to identify an initial set of climate risks for that system. This initial list of risks was then used as input into 7 online system workshops held during September 2023. The format of these workshops is outlined in Figure 2. In each workshop, there were 25–35 participants, representing all levels of government, statutory authorities, advocacy groups, non-governmental organizations, research sector, peak bodies, and industry.

After the online system workshops, a face-to-face integration and synthesis workshop was held in Adelaide, South Australia, in October 2023. This brought together key stakeholders to examine the risks from all of the systems and considered how the risks mapped to a larger set of cross-systems risks. The workshop had 51 participants, with 90% of those having attended an online system workshop and being familiar with the NCRA first pass process. A final list of 56 nationally significant risks and 12 priority risks were identified. The priority risks were then provided to the Australian government for further consultation, resulting in the final selection of 11 priority risks^29^ as input into the second pass quantitative assessment of the NCRA conducted during 2024.

An exception was made for the First Nations Values and Knowledge system, where instead of an online workshop, a more culturally appropriate face-to-face workshop process was developed. An expression of interest to participate in the study was circulated through researchers’ networks to First Nations Peoples across a diversity of groups from around Australia. The aim was to have representation from all states, with a combination of urban and regional areas, and bioregions (e.g., desert, sea country, freshwater, rainforest, urban areas, and Torres Straits). First Nations People who attended the other 7 system workshops were also invited to participate. The Australian government circulated the expression of Interest to contacts in the Torres Strait Islands. Where gaps in representation were identified, for example, in urban areas (e.g., Melbourne, Darwin, and Sydney), the relevant Native Title Body was contacted to extend an expression of interest.

Workshops were held in November 2023 in Brisbane, Queensland and Adelaide, South Australia, to provide opportunity for Traditional Owners from across the country to attend. In total, 23 First Nations stakeholders came together in these workshops to discuss climate change impacts on First Nations values and knowledge and to review the priority risks.

## Review of other national climate risk assessments

To understand the process and outcomes of other national climate risk assessments from around the world, a desktop review was undertaken. Fifteen documents were identified for review from 13 countries or regions around the world (Table 1). These documents were labeled as national climate risk assessments or contained similar content (e.g., national climate change impacts).

**Table 1:** Countries and documents considered in the review of climate risk assessments

Country or region	Report name	Document type	Iteration number; year of publication	Reference
UK	UK Climate Change Risk Assessment: Government Report 2012	National Climate Risk Assessment	1st; 2012	His Majesty’s Government (2012)^30^
UK	UK Climate Change Risk Assessment 2017	National Climate Risk Assessment	2nd; 2017	His Majesty’s Government (2017)^31^
UK	The Third UK Climate Change Risk Assessment 2022	National Climate Risk Assessment	3rd; 2022	Richard et al.^19^
Europe	European Climate Risk Assessment	National Climate Risk Assessment	1st; 2024	European Environment Agency (2024)^18^
Germany	Climate Impact and Risk Assessment 2021 for Germany	National Climate Risk Assessment	2nd; 2021	Walter Kahlenborn et al.^20^
Finland	Finland’s Eight National Communication under the United Nations Frameworks Convention on Climate Change	National Climate Risk Assessment	8th; 2022	Ministry of the Environment and Statistics Finland (2022)^32^
Canada	Canada in a Changing Climate: Advancing our Knowledge for Action	National Climate Risk Assessment	4th; 2017^a^	Government of Canada (2019)^33^
US	Fifth National Climate Assessment	National Climate Risk Assessment	5th; 2023	Jay et al.^21^
New Zealand	National Climate Change Risk Assessment for Aotearoa New Zealand: Main report – Arotakenga Tūraru mō te Huringa Āhuarangi o Āotearoa: Pūrongo whakatōpū	National Climate Risk Assessment	1st; 2021	Ministry for the Environment (2020)^23^
Japan	Assessment Report on Climate Change Impacts in Japan	National Climate Risk Assessment	2nd; 2020	Ministry of the Environment - Japan (2020)^34^
Singapore	Singapore’s Third National Climate Change Study	Climate Change Study	3rd; 2024	Center for Climate Research Singapore (2024)^35^
Nepal	Vulnerability and Risk Assessment Framework and Indicators for National Adaptation Plan (NAP) – Formulation Process in Nepal	Framework	1st; 2017	Ministry of Population and Environment(MoPE) (2017)^36^
Vanuatu	Vanuatu rapid climate risk assessment framework and methodology	Framework	1st; 2023	Beca International Consultants Ltd (2023)^37^
South Africa	A co-produced national climate change risk and vulnerability assessment framework for South Africa	Framework	1st; 2023	Ziervogel et al.^38^
Brazil	National Adaptation Plan to Climate Change	Adaptation plan	1^st^; 2016	Ministry of Environment (2016)^39^

a Canada launched its latest report in 2017. However, they release chapters continuously, with the last one released in 2024.

Table 1 provides information regarding the documents reviewed for the different nations or regions. We aimed to include one risk assessment for each continent in this review, and at least one country with many iterations of their process.

During the review, we looked for common themes aligned with our experience when undertaking the first pass of the Australia’s NCRA, and developed a list for information to collect and compare across the various documents. This information includes aspects of the assessment objective, model (e.g., mandated by law), methodology (engagement method, limitations), timing (iteration number, frequency), risk characteristics (systems area, categories of risks, time periods, uncertainty), the inclusion of Indigenous knowledge, the inclusion of complex or systemic risks, and the types of lessons learned.

In our attempt to conduct a systematic review, we found there were few fully developed risk assessments for which priority risks had been developed to inform policy and decision-making. Rather, the risk assessments had different audiences and goals. Some were developed with the goal of describing possible methodologies for future climate risk assessments. For example, the Nepal,^36^ Vanuatu,^37^ and South African^38^ documents describe a proposed methodology. However, no accompanying NCRA has been completed or published yet for South Africa, and Vanuatu’s methodology has only been applied to one case study focused on tourism.^40^ Other documents, such as those from Singapore,^35^ focused on climate science and impacts, including the application of climate projections and scenario modeling. Japan’s climate change assessment went a step further, describing the chain of consequences from each climate/natural factor for 7 sectors and their flow-on effect across other sectors.^34^ Finally, there were other risk assessments that went well beyond climate impact, identifying climate risks to inform policy, such as the assessment undertaken by Europe.^18^

Each risk assessment was also at different cycles or iterations of delivery and implementation, with some countries starting their first assessment while others had conducted several cycles (e.g., 5th iteration for the US, 3rd for the UK, and 4th for Canada). Some risk assessments were short and fed into other stages of the process (e.g., first pass of Australia’s NCRA), while others had longer time frames and were self-contained (e.g., Germany). Because of this difference in iterations, there are differences in the maturity and level of detail expressed or analyzed within the assessments. We found at the country level, assessments could also change across cycles because they incorporated learnings from a previous cycle, resulting in adjustments to the methodology or focus. Therefore, even within a country, it can be somewhat difficult to compare risk assessments from cycle to cycle. However, learnings from each cycle are important, and the implementation of these learnings in subsequent cycles shows a growing level of maturity.^41^

Because of these differences (e.g., objectives, intended use to inform decisions, maturity levels, time frames, etc.), the documents collected were not directly comparable in their results. Even assessments undertaken by a single organization, such as the World Bank Group,^42^ which has undertaken high-level assessments of physical climate risks for 70+ countries worldwide, have variations across nations in how they report impacts. For example, Mongolia^43^ and Bulgaria’s^44^ assessments both discuss the country’s climatology, climate-related natural hazards, impacts and adaptation information. However, Bulgaria does not consider community impacts and focuses on different sectors of the economy compared to Mongolia. Such flexibility is often needed so that each nation can tailor its assessment for its own situation and context. The experience of each country in undertaking its climate risk assessment is a rich opportunity to garner learning and insights to improve ongoing processes across other countries. As each country follows a slightly different process to respond to its own needs and contexts, this allows other countries to see what would benefit them when planning their climate risk assessment or next iteration. In this vein, we identified 4 themes relating to the process from the experience of the first Australian NCRA that we believe were foundational to its success. We compared these themes with descriptions of processes undertaken by other nations. This review provides an important first look at understanding the range of national climate risk assessments that have been undertaken around the world, identifying key similarities and differences for consideration in future climate risk assessments.

## Process insights

From our review of international approaches and reflecting on our own experiences with the process to develop the first pass of Australia’s NCRA, we outline 4 key themes of process insights. Each theme has benefits and limitations as discussed in the context of the Australian experience and contrasted with insights from other national climate risk assessments around the world.

### A rapid iterative staged approach

The Australian NCRA took a deliberately iterative approach that allowed for iterations within the first pass and second pass assessment process, as well as allowing for adjustments in future iterations of the national assessment. Other countries have taken a similar staged approach in the release of their information as part of their NCRA. For example, in 2019, Canada^33^ released the first *Canada’s Changing Climate Report*,^22^ in which a series of authoritative online reports that focus on impacts, adaptation, and resilience were released at different time intervals (at the rate of about one a year). While the process for gathering and releasing Canada’s full climate risk assessment is expected to last about 6 years, having a staged approach enables the assessment to be released and updated based on available information.

The Australian NCRA, which is expected to be repeated, had a very short time frame to complete the first pass, with 4 months for the stakeholder engagement process.^8^ This was also the case for New Zealand, whose time frame was 9 months (including all stages of the process). On the other hand, Germany’s assessment took 3 years to release and is expected to be repeated every 6 years. Many reasons can explain these different time frames, but often, the timing corresponds to policy or election cycles. This is the case for the European Climate Risk Assessment (EUCRA),^18^ which took 18 months (including a description of the risks as well as their quantitative analyses). The timing and duration correspond with the elections to the European Parliament.

Having a rapid, iterative, and staged approach has many advantages.^41^ The staged approach enables information to be released throughout the process to facilitate rapid knowledge sharing and increase transparency and stakeholder input. The staged approach also ensures that the resulting information can be used by other processes that occur in parallel. This is the case for the development of Australia’s National Adaptation Plan,^45^ as well as regional and local risk assessments or adaptation plans that may not require the output from the national quantitative analysis, but can benefit from the framing and ranking of the risks within the national context.

While implementing the first pass of the NCRA over such a short time frame was resource intensive, it had the advantage of keeping the stakeholders, who had direct input in eliciting the risks, engaged throughout the different steps of the process (see Figure 1). This allowed for a coherent overall assessment of the risks across systems. Such a rapid, staged approach will also help to repeat this process on a more regular basis as new information becomes available and adaptation plans are put into place, which might shift the priority of the previously identified risks.

Working over such a short time frame, however, limited the time available for the literature review. This limitation was compensated through the expert elicitation, which despite being resource intensive, allowed for the initial list of risks to be refined and validated in a short period of time.

### Participant diversity

One of the broad goals of the first pass of the Australian NCRA was to develop risks not only based on the literature review but also to have deep engagement with stakeholders across different systems and levels of government. The aim was to have the stakeholders refine and validate the climate risks initially identified through the literature review based on their knowledge and experience, and to prioritize the risks in terms of impact and urgency. This participatory approach enabled the development of a shared understanding of the national values that are at risk from climate change and that cut across traditional sectoral and jurisdictional boundaries. Such an approach is often used in climate risk assessments as it ensures the process is relevant for the decision-making context^46^ and helps identify knowledge gaps in climate policy development.^47^ A diversity of stakeholders was therefore necessary to address cross-system risks and to think through risks systemically and across the boundaries of traditional management.^48^^,^^49^

In reviewing other country’s national climate risk assessments, we found that information on stakeholder engagement processes is often scarce. Many reports indicate that stakeholder engagement took place, or the process included engagement, but the method and format of how this occurred were not fully explained. The US NCRA^21^ was one of the few examples that does elucidate the engagement that occurred. It states that over 7,000 people registered to be part of the climate risk assessment review, and over 2,000 people participated by providing review comments on the draft reports. However, there is no indication that there were workshops or other types of expert elicitation processes undertaken or whether there was a diversity of experiences represented in the comments collated on the draft reports.

Overall, our review of climate risk assessments shows that it is challenging to have significant stakeholder engagement within the risk identification and prioritization process. This is because stakeholder engagement is a time-consuming and relational process that asks stakeholders to give up their time to contribute to the assessment and engage effectively. Having the time and resources to engage with stakeholders can be useful for developing a coalition of organizations and communities willing to work through climate risks over time and think through adaptation management and how it can be implemented at the regional or local scale. This can be very valuable if there is the ability to bring people together.^41^ Finally, having a diverse representation of stakeholders involved in the climate risks assessment process is critical to ensure buy-in and capture the views and risks important to those on the ground dealing with the impacts of climate change. One limitation of the first pass of the Australian NCRA, however, is the potential to miss out on some stakeholder perspectives because of the smaller base of participants involved in the participatory approach, as opposed to a larger engagement such as the one chosen by the US.

### First Nations involvement

There is growing evidence of the importance of First Nations knowledge in guiding climate adaptation planning,^50^ especially as it pertains to understanding the context of local climate impacts and potential adaptation options.^51^ It is also recognized, however, that there is a gap in how First Nations knowledge is incorporated into national climate risk assessments.^52^^,^^53^ Few assessments, including the first pass of the Australian NCRA had the time and resources to design and sufficiently complete a culturally appropriate engagement with First Nations peoples.

Further engagement is required to work through the climate risks across the various First Nations communities around Australia and how those risks differ from region to region and within different communities. It was also acknowledged that where Traditional Ecological Knowledge is incorporated into risk identification and management, issues around Indigenous intellectual property would need to be discussed and agreed upon before publication in government reports or scientific literature. The inclusion of Indigenous Cultural and Intellectual Property (ICIP) rights and the importance of indigenous-led processes in future stages of the Australian NCRA was a key message that came out of workshops with First Nations peoples. For any co-produced work, it will be critical to establish rigorous processes to ensure these rights are protected,^54^ and discussions of governance of land and knowledge to protect and adapt management to climate change must be part of the discussions on climate change risks to First Nations peoples.^55^^,^^56^

The importance of Indigenous engagement and incorporating Indigenous knowledge and values in national climate risk assessments is not unique to Australia. In our review, we found several other countries have also attempted to incorporate Indigenous knowledge into their national climate risk assessments in various ways. While many assessments recognize the importance of engaging Indigenous stakeholders,^21^^,^^22^^,^^23^ they often provide little detail on how the engagement occurs or if the engagement with Indigenous stakeholders successfully incorporated Indigenous knowledge and perspectives into the risk assessment process and identification of priority risks.

How Indigenous risks are brought into a NCRA can vary widely. In the Finland assessment report,^32^ the authors discuss how climate change risks impact the Sami people, but the assessment does not outline how this knowledge was brought into the report or if Indigenous groups were engaged. The New Zealand assessment^23^ presents more detailed information about how Indigenous communities were engaged, which included a *hui*, which is a meeting of a group of Indigenous people who come together to discuss.^57^ New Zealand had originally planned 4 group meetings but was only able to accomplish one in the time frame of the assessment (9 months). More recently, a new report^58^ released as part of Canada’s National Knowledge Assessment process is dedicated to Indigenous risks and is written from the perspective of First Nations Inuit and Metis living in what is currently known as Canada. Specific details about the authorship team, engagement process, and key principles underlying the report are provided. This is the most comprehensive report of this nature that we reviewed in terms of reporting on climate change risks, experiences, and approaches from Indigenous perspectives.

### Co-development and learning

The rapid and iterative process of the first pass of Australia’s NCRA allowed for several rounds of co-learning and co-development to occur through discussions in small breakout groups and larger plenary conversations. Each iteration allowed the articulation of risks to be adjusted and refined to better reflect the experience and knowledge of that risk contributed by diverse stakeholders. Observations about this process of learning are provided in a related paper.^41^

In most of the national climate risk assessments we reviewed, risks were not co-developed with stakeholders. The literature also points to limited stakeholder coordination and interaction in climate risk assessment processes in general.^17^ The concerns are that limited engagement with stakeholders can constrain discussions and understanding of risks. In most cases, risks were identified and developed through literature review,^19^ while in other cases risks were only reviewed through stakeholder consultation, review groups or advisory groups, rather than being co-developed.^21^^,^^33^ As such, co-development of priority climate risks and co-learning about climate risk and adaptation through the risk assessment process is a unique aspect of the first pass of the Australian NCRA. Running such co-development process is resource-intensive, and required additional time, as well as emotional and relational skills for working with large groups. It also required the ability to rapidly draw from established networks across various sectors so that experts could come together at short notice. Finally, a participatory research approach also requires care around managing inherent subjectivity of some of its elements, and transparency about the judgment of the research team or the stakeholders involved. Such subjectivity was minimized using a range of strategies including the careful selection of system experts as well as stakeholders, equal access to the information collected and shared, and equal opportunities to participate and contribute to this process. Designing and delivering such processes can be complex and necessitated the involvement of experienced and professional facilitators.

In the Australian context, the participatory process and expert facilitation used to implement the first pass of the NCRA uncovered important cross-system risks. In each of the online system workshops, stakeholders were stepped through a process where they could see how the risks from their systems could impact other systems, and how the risks in other systems could impact their system. Cross-cutting issues, commonalities, and challenges that were noted within the online systems workshops helped guide the research team to identify a set of cross-system risks.

A focus on systemic risks was also seen in several other national climate risk assessments. For example, in the European Union Climate Risk Assessment,^18^ the 36 risks developed during the assessment were grouped into 5 interrelated risks. These systemic and cross-cutting risks were then described through storylines. In the second UK Climate Change Risk Assessment,^31^ cross-cutting risks were introduced that allowed for a more systems-based analysis of national climate risks. This was primarily addressed in the cross-cutting issues section of the assessment report and focused on interactions between domain risks (e.g., natural environment risks, water and soil condition, and agricultural productivity impacts) and their impact on adaptation planning and response. They noted data on systemic risk is not collected across domains, posing a substantial barrier to understanding cross-cutting issues when undertaking the risk assessment process.

## Discussion

Comparing the process used to implement the first pass of Australia’s NCRA with processes used by other countries and/or regions allows us to both learn from their experience and consider how national climate risk assessments can be improved globally. This will be particularly relevant as Australia considers future cycles and iterations of its NCRA. We found countries that have conducted multiple cycles of their climate risk assessment tended to adjust their methodology as they perform the next round of their risk assessments.^19^^,^^21^^,^^32^ Adjustments are likely made to capture aspects missing in a previous round, adjust to different timelines or needs, and/or to incorporate information that was not previously available. For example, the UK has reduced their scope over time. Between the first and second iterations of their climate risk assessment, the UK team reduced the number of risks reported, conducted less stakeholder engagement, focused on “urgency” classification, and had a much less data-driven process with less focus on risk categorization, classification and ranking.^30^^,^^31^ On the other hand, the US added a chapter in their fifth assessment dedicated to sector interaction, multiple stressors and complex systems that consider interdependencies, and compounding and cascading impacts.^21^ Canada also updated its output in its fourth assessment by adding a chapter dedicated to Indigenous risks, written from the perspective of Indigenous peoples.^58^

One aspect starting to be addressed in national climate risk assessments that have had multiple rounds of assessment is the inclusion of transition risks. Transition risks are the risks associated with transitioning to a lower-carbon economy, which may require policy, legal, technology, and market changes to address mitigation and adaptation requirements related to climate change.^59^ Transition risks were out of scope for the Australian NCRA.^8^ Other risk assessments, such as those from the European Union (EU) and New Zealand, have also not included transition risks as they are placed under a different policy mechanism and regulatory framework from that of climate change risk, such as a net zero policy or a renewable energy program. Separating transition risks from physical climate risks is difficult, however, as they are heavily intertwined, and both are necessary parts of climate mitigation and adaptation planning. Transition risks compound with physical climate risks through time, impacting not only how risks manifest but how they are prioritized for climate mitigation and adaptation action.^60^^,^^61^ In their fifth climate risk assessment, the US contains a key message in their Social Systems and Justice chapter^62^ where they explicitly mention transitions in terms of the risks they pose to deepening injustices from the impact of climate change, with calls for societal transitions, such as to renewable energy, to be equitable.

Another important aspect for national climate risk assessments and their subsequent cycles identified in our review is the need to monitor and evaluate the process and its outcomes. Monitoring and evaluation of the risk assessment and how that might have impacted actions on the ground is important to understand which parts of the process worked, their impact, how they created change, and which parts need to be improved.^63^^,^^64^ This can inform adjustments to the methodology for the next risk assessment cycle to ensure gaps are filled and to take advantage of identified opportunities.^63^ None of the risk assessment processes that were reviewed, except for Brazil^39^ although it is an adaptation planning document, indicated the implementation of a monitoring and evaluation process. Many reports, however, discuss the importance of monitoring in order to assess the efficacy of adaptation actions for future iterations of the risk assessment. In the EU climate risk assessment, suggestions for action included changes in legislation, monitoring, co-funding, and technical support to improve the analysis of major climatic risks. They noted that a third of the 36 major climate risks identified required more research, better monitoring, or a review of the policy framework for further assessment. Some legislation and regulatory policies already have monitoring embedded in the policy, providing a platform and process that can support more efficient data collection. Many assessments indicate that other parts of the government should monitor the risks, but they do not elucidate how this will happen or how it will be resourced. One example that does, however, is in the Brazil assessment, where evaluation has been embedded into the process with a four-year cycle for climate adaptation planning and the last year of each cycle dedicated to formal evaluation.^65^

During the first pass of Australia’s NCRA, it also became apparent that a qualitative and bottom-up process was needed to complement the data compiled through the literature review in order to identify values at risk and develop the risks in a coordinated and co-developed fashion.^66^^,^^67^ While the first pass started with a data-driven approach, with specific hazards and time-scales at which to consider the risks, the stakeholders challenged the need to interrogate the scientific climate data during the online system workshops; instead, they moved on quickly to discussing risks in their system, including the compounding impacts of multiple hazards on assets and communities.^41^ This was perhaps a symptom of the limited time available for the process or that stakeholders already acknowledge that impacts from the hazards are already being felt, and what it will look like in 2050 or 2090 matters less than what needs to be done to address risks now. Discussing values that are at risk now is more urgent for many stakeholders.

A final consideration based on this review of national climate risk assessments, is whether there is merit in the development of a common global framework to provide greater consistency and comparison of risk assessment process and outputs among countries. This is an approach that is being developed by several sectors. For example, the Electricity Sector Climate Information project in Australia has developed a climate risk assessment method to help the electricity sector consider climate risk alongside other business risks.^68^ The Australian Public Sector has also defined frameworks for its organizations to assess climate risks and opportunities,^69^ and many Australian companies are considering their climate-related risks according to the Taskforce for Climate-Related Financial Disclosures (TCFD).^70^ Adopting a common framework and methodology at the global scale could lead to greater coordination in responding to climate risks that emerge as international priorities, especially as many climate risks extend beyond national borders. For some risks there is already a strong recognition of the need to think at the global scale. For example, global processes such as supply chains and telecommunications affect the ability of citizens to receive goods and services and are vulnerable to climate change impacts. This highlights the potential value of greater international coordination and improved consistency in the development and implementation of national climate risk assessments and adaptation plans. The IPCC is the leading international body for assessing the global impacts of climate change,^71^ but could benefit from more direct and active multilateral collaboration among countries advancing national climate risk assessments.

### Conclusion

Experience from the process of implementing the first pass of Australia’s NCRA coupled with our review of national climate risk assessments undertaken by other countries highlight that cross-system, national-level assessments are important to prioritize risks and adaptation responses. It is essential that diverse stakeholders are involved in the risk assessment process, particularly those responsible for risk management and adaptation planning. The delivery of national climate risk assessments using co-development processes with stakeholders is not common nor is the culturally appropriate engagement of First Nations peoples and the incorporation of Indigenous knowledge and values. Following our review of 15 documents on the process and outcomes of national climate risk assessments from around the world, there is much that can be learnt from the different approaches and experiences of different countries. The science and quality of national climate risk assessments could be advanced through the development of a common framework for collaboration among nations undertaking national climate risk assessments. This could also improve understanding of global climate risks and interactions, identifying critical interdependencies, and how risk ownership and governance can be managed across borders.

## Resource availability

### Lead contact

Requests for further information and resources should be directed to and will be fulfilled by the lead contact, Fanny A. Boulaire (fanny.boulaire@csiro.au).

### Materials availability

This study did not use or generate any material.

### Data and code availability

• This paper analyses existing, publicly available reports, accessible at https://doi.org/10.25919/64vf-f754, https://doi.org/10.25919/pcgz-w734, https://doi.org/10.25919/bps2-ss65, https://doi.org/10.25919/pdk7-c967, https://doi.org/10.25919/0teh-6257, https://doi.org/10.25919/d1h1-tg19, https://doi.org/10.25919/4btv-aj14, https://doi.org/10.25919/ee1e-sc33, https://doi.org/10.25919/7np9-9277, https://doi.org/10.25919/w7qa-2g07.
• This paper does not report original code.
• Any additional information required to reanalyze the data reported in this paper is available from the lead contact upon request.

## Acknowledgments

We recognize the First Peoples of Australia and their ongoing connection to culture and Country. We acknowledge Aboriginal and Torres Strait Islander peoples as the Traditional Owners, Custodians and Lore Keepers of the world’s oldest living culture and pay respects to their Elders past and present. We would like to thank everyone who participated in the workshop processes for the first pass of Australia’s NCRA, contributing their time and expertise to the discussions. This project received Ethical Clearance No. 126/23 from the CSIRO Social and Interdisciplinary Science Human Ethics Research Committee. It complies with the requirements of Australia’s National Statement on the Ethical Conduct in Human Research.

## Author contributions

Conceptualization, F.A.B. and B.B.L.; methodology, F.A.B., B.B.L., and S.C.; investigation, F.A.B., S.C., A.F., L.R., T.C., M.P., R.D., and B.B.L.; writing – original draft, F.A.B., S.C., and B.B.L.; writing – review & editing, A.F., L.R., T.C., M.P., R.D., G.B., and S.B.; funding acquisition, B.B.L. and G.B.

## Declaration of interests

CSIRO is a partner in the Australian Climate Service (ACS) and was responsible for technical delivery of the first pass of the NCRA, led by Australian Government Department of Climate Change, Energy, the Environment and Water (DCCEEW) and the ACS.
